# The status of low anterior resection syndrome: data from a single-center in China

**DOI:** 10.1186/s12893-023-02008-4

**Published:** 2023-05-06

**Authors:** Jing Su, Qianhui Liu, Dagui Zhou, Xiaofeng Yang, Guiru Jia, Lijun Huang, Xiao Tang, Jiafeng Fang

**Affiliations:** 1 Department of Nursing, the Third Affiliated Hospital of Sun Yat-sen University, Guangzhou, China; 2 Department of Gastrointestinal Surgery, the Third Affiliated Hospital of Sun Yat- sen University, Tianhe Road 600, 510630 Guangzhou, China

**Keywords:** Low anterior resection syndrome, Rectal neoplasms, Bowel dysfunction, Quality of life, Satisfaction survey

## Abstract

**Aim:**

The incidence and risk factors of low anterior resection syndrome (LARS) largely variate in different studies. In addition, there is lack of study on how patients evaluate the therapeutic effect of LARS. This single-center retrospective study aims to investigate the status of LARS in Chinese patients undergoing laparoscopic low anterior resection (LAR).

**Methods:**

Consequent patients undergoing laparoscopic LAR and free from disease recurrence from January 2015 to May 2021 were issued with both LARS questionnaire and satisfaction survey. Related data were collected and analyzed.

**Results:**

Both LARS questionnaires and self-made satisfaction survey were received from 261 eligible patients. The overall incidence of LARS was 47.1% (minor in 19.5%, major in 27.6%), decreased with the passage of postoperative time (64.7% within 12 months, and 41.7% within 12–36 months), and became stable 36 months later (39.7%). The most common symptoms were defecation clustering (n = 107/261, 41.0%) and defecation urgency (n = 101/261, 38.7%). According to the multivariable regression analysis, risk factors of major LARS were: 1 year increase in age (OR 1.035, 95% CI 1.004–1.068), protective stoma (OR 2.656, 95% CI 1.233–5.724) and T_3 − 4_ stage (OR 2.449, 95% CI 1.137–5.273). Most patients complained defecation disorder (87.3%) to doctors and 84.5% got suggestions or treatments for it. However, only 36.8% patients thought the treatments worked for them.

**Conclusions:**

LARS frequently occurs after laparoscopic LAR, while the therapeutic effect is not satisfying. Elder, advanced T-stage and protective stoma were risk factors for postoperative major LARS.

## Introduction

Colorectal cancer is one of the third most common malignant tumors in the world and it even ranks 2nd in China [1, 2]. With the great progress of surgery and adjuvant therapy, the therapeutic outcome of colorectal cancer has been largely improved in the past decades. Thus, postoperative quality of life (QoL) has drawn more and more attention of either doctors or patients [3, 4]. For rectal cancer, one of the most common complications largely impacting QoL is bowel dysfunction, named as low anterior resection syndrome (LARS), and manifested by a broad spectrum of symptoms, including incontinence for flatus and/or liquid stool, clustering of stools, fecal urgency, and an extremely high or low frequency of bowel movements [5].

Previous assessments of LARS have been largely limited due to lack of standardised instruments. In 2012, the LARS score was firstly described to measure bowel dysfunction after rectal cancer surgery [6]. The score is closely related with status of postoperative QoL and thus has been widely translated and applied to assess the bowel function after low anterior resection (LAR) [7, 8]. Previous studies revealed that the incidence of LARS variated from 50 to 90% of patients undergoing LAR [5, 9]. In addition, the exact mechanism and risk factors of major LARS also largely variate in different studies [10, 11].

Considering that the patient characteristics, therapeutic methods and effects might differ with time and in different countries, this study aims to evaluate the incidence and risk factors of LARS and therapeutic effects in Chinese patients undergoing LAR from a single center. The surgery was performed by a same surgical team, with a same anastomotic method (straight colorectal/coloanal anastomosis) and cases of severe complications such as postoperative anastomotic leakage were excluded, in order to better figure out objective perioperative risk factors of major LARS and thus we can apply more early and active intervention for these patients for better postoperative QoL.

## Methods

### Study design

This was a retrospective study including consequent patients undergoing radical laparoscopic LAR and free from disease recurrence in a single Chinese hospital. Eligible participants were issued with LARS questionnaire (Chinese version) and self-made satisfaction survey scale by phone or face to face interview. The questionnaire and patient data were collected and analyzed to figure out the incidence and risk factors of LARS. This study was approved by the Ethics Committee of the Third Affiliated Hospital, Sun Yat-Sen University.

### Eligibility and exclusion criteria

Patients diagnosed as rectal cancer and underwent radical laparoscopic LAR and straight colorectal/coloanal anastomosis (with or without a protective stoma) by a same surgical team in Department of Gastrointestinal Surgery of the Third Affiliated Hospital of Sun Yat-sen University from January 2015 to May 2021 were eligible. Exclusion criteria were either local or distal recurrence, neoadjuvant radiotherapy, severe complications such as postoperative anastomotic leakage, failure of protective stoma closure, data missed and no responding to LARS questionnaire. Informed consent was obtained from all included patients in this study.

### Variables

The LARS questionnaire contains a 5-item scoring system to evaluate the bowel function in patients after LAR for rectal cancer. The total LARS score ranges from 0 to 42 points and patients were classified as three ordinal scale, containing no LARS (0–20 points), minor LARS (21–29 points) and major LARS (30–42 points). The satisfaction survey contains 6 questions and patients will need answer either part or all of them according to their individual status.

### Data

Data of eligible patients were collected, containing patient demographics (age, sex, body mass index), cancer characteristics (pathological T-stage, N-stage, tumor level above annal verge), neoadjuvant therapy (none or neoadjuvant chemotherapy), comorbid status (hyperlipemia, diabetes) and whether a protective stoma was performed. Postoperative data included length of follow-up (time from operation or stoma closure to LARS assessment).

### Statistical analysis

Statistical analysis was performed using the SPSS 25.0 statistical software (IBM Corp. USA). The data were expressed as mean ± standard deviation (SD) for continuous variables and number for categorical variables. Variables of potential risk factors for major LARS were compared with the t-test or Chi-square test, and variables with *P* value no greater than 0.25 were fitted in a multivariable logistic regression model. The logistic regression model was selected to estimate associations between variables and major LARS, with results expressed as adjusted odds ratios (OR) with 95% confidence intervals (CI), and *P* < 0.05 was considered significant.

## Results

### Overall incidence and severity of LARS

Totally 261 cases of LARS questionnaires were received. The median time from surgery to LARS assessment was 28.1 months (range 1–74 months). The overall incidence of LARS was 47.1% (n = 123/261). The incidence of minor and major LARS was 19.5% (n = 51/261) and 27.6% (n = 72/261), respectively.

### Incidence and severity of LARS in different postoperative time

**Fig. 1:**
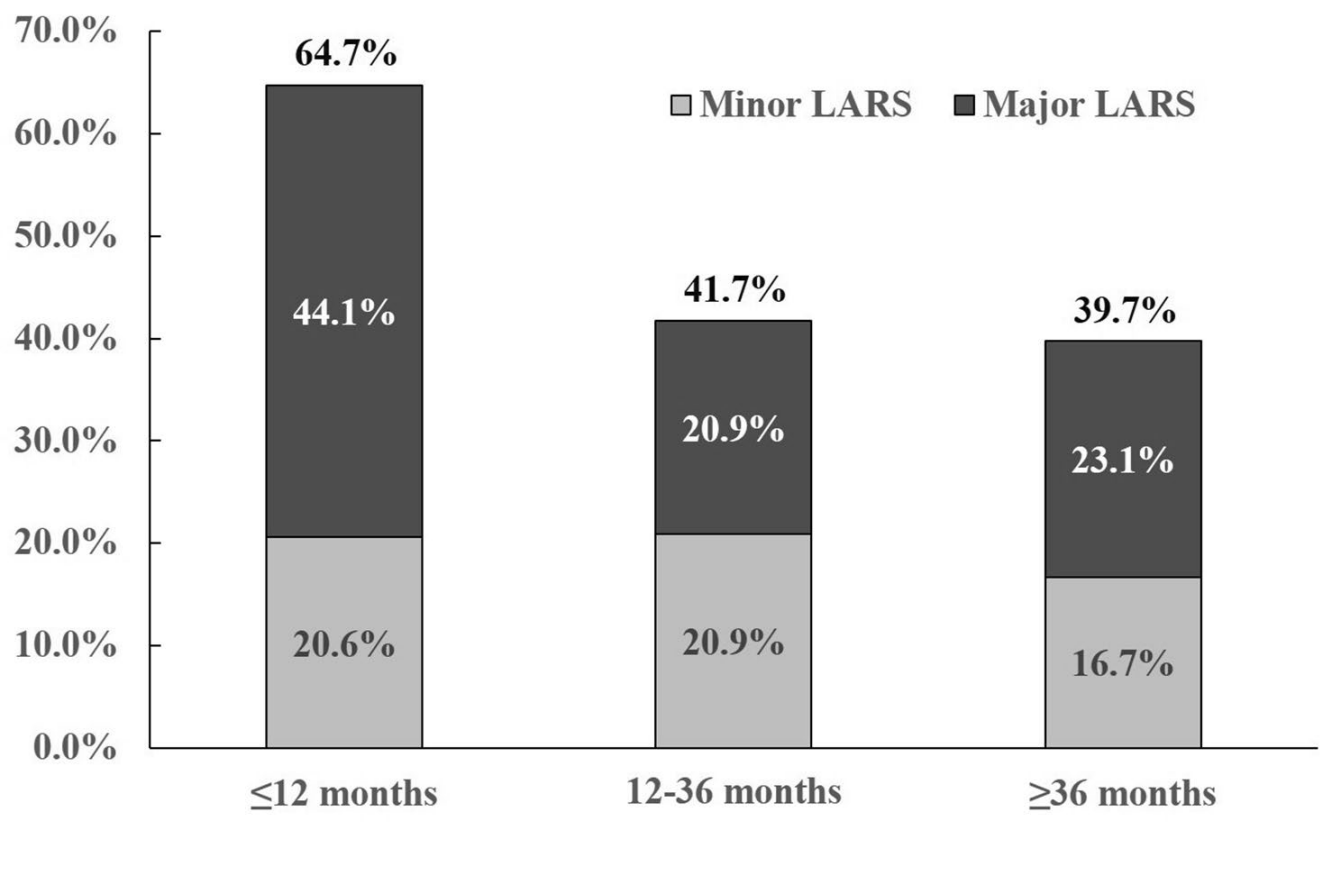
The incidence and severity of LARS in three groups of different time to assessment. The incidence of LARS within 12 months postoperatively was 64.7%, and decreased significantly within 12–36 months (41.7%, *P* = 0.002), especially incidence of major LARS (44.1% vs. 20.9%). Compared with that within 12–36 months, the incidence of LARS became stable 36 months postoperatively (39.7%, *P* = 0.754)

Basing on postoperative time to LARS assessment, the patients were divided into three groups: within 12 months, within 12–36 months and 36 months later. The basic patient characteristics and perioperative data were shown in Table 1. There was no significant statistical difference in these variables among these three groups. The incidence and severity of LARS in three groups were shown in Fig. 1. The incidence of LARS within 12 months postoperatively was 64.7%, and decreased significantly within 12–36 months (41.7%, *P* = 0.002), especially incidence of major LARS (44.1% vs. 20.9%). Compared with that within 12–36 months, the incidence of LARS became sTable 36 months postoperatively (39.7%, *P* = 0.754).

**Table 1:** Patient characteristics and perioperative data at different time to LARS assessment

Variable	Postoperative time to LARS assessment (months)			*P* value
	≤ 12 (n = 68)	12–36 (n = 115)	≥ 36 (n = 78)	
Sex				0.508
Male	42	74	55	
Female	26	41	23	
Age (year)	62.22 + 11.39	61.26 + 10.09	59.88 + 11.85	0.431
<60	28	43	32	0.831
≥60	40	72	46	
BMI (kg/m^2^)	22.58 + 2.96	22.54 + 3.57	23.07 + 3.27	0.519
T-stage				0.453
T_1 − 2_	24	44	23	
T_3 − 4_	44	71	55	
N-stage				0.541
N_0_	46	81	49	
N_1 − 2_	22	34	29	
Tumor height				0.152
≤5 cm	17	35	18	
5-10 cm	44	56	42	
≥10 cm	7	24	18	
Total mesorectal excision				0.523
Yes	18	35	18	
No	50	80	60	
Excision excision				0.988
Yes	2	3	2	
No	66	112	76	
Hyperlipemia				
Yes	4	12	6	0.542
No	64	103	72	
Diabetes				0.889
Yes	9	16	9	
No	59	99	69	
Protective stoma				
Yes	11	22	17	0.642
No	57	93	59	
Neoadjuvant therapy				
Yes	27	40	21	0.251
No	41	75	57	

The score components of LARS in three different groups were shown in Table 2. Totally 30.3% patients could not control flatus (n = 79), 28.0% patients suffered from liquid stool leakage (n = 73), 45.2% patients presented abnormal frequency of open bowels (n = 118), the incidence of open bowels within 1 h and strong urge to open bowels was 58.6% (n = 153) and 53.6% (n = 140), respectively. Taking together, the most common symptoms were defecation clustering (n = 107, 41.0%) and defecation urgency (n = 101, 38.7%).

**Table 2:** Incidence of LARS and score components at different time to LARS assessment

Variable	Postoperative time to LARS assessment (months)			Total (n = 261)
	≤ 12 (n = 68)	12–36 (n = 115)	≥ 36 (n = 78)	
LARS				
No LARS	24 (35.3%)	67 (58.3%)	47 (60.3%)	138 (52.9%)
Minor LARS	14 (20.6%)	24 (20.9%)	13 (16.7%)	51 (19.5%)
Major LARS	30 (44.1%)	24 (20.9%)	18 (23.1%)	72 (27.6%)
Cannot control flatus				
No, never	39 (57.4%)	88 (76.5%)	55 (70.5%)	182 (69.7%)
Yes, less than once per week	16 (23.5%)	5 (4.3%)	3 (3.8%)	24 (9.2%)
Yes, at least once per week	13 (19.1%)	22 (19.1%)	20 (25.6%)	55 (21.1%)
Liquid stool leakage				
No, never	46 (67.6%)	87 (75.7%)	55 (70.5%)	188 (72.0%)
Yes, less than once per week	12 (17.6%)	5 (4.3%)	3 (3.8%)	20 (7.7%)
Yes, at least once per week	10 (14.7%)	23 (20.0%)	20 (25.6%)	53 (20.3%)
How often open bowels (24 h)				
Less than once per day	6 (8.8%)	8 (7.0%)	3 (3.8%)	17 (6.5%)
1–3 times per day	27 (39.7%)	69 (60.0%)	47 (60.3%)	143 (54.8%)
4–7 times per day	20 (29.4%)	25 (21.7%)	23 (29.5%)	68 (26.1%)
More than 7 times per day	15 (22.1%)	13 (11.3%)	5 (6.4%)	33 (12.6%)
Open bowels within 1 h				
No, never	15 (22.1%)	56 (48.7%)	37 (47.4%)	108 (41.4%)
Yes, less than once per week	19 (27.9%)	12 (10.4%)	15 (19.2%)	46 (17.6%)
Yes, at least once per week	34 (50.0%)	47 (40.9%)	26 (33.3%)	107 (41.0%)
Strong urge to open bowels				
No, never	22 (32.4%)	60 (52.2%)	39 (50.0%)	121 (46.4%)
Yes, less than once per week	7 (10.3%)	17 (14.8%)	15 (19.2%)	39 (14.9%)
Yes, at least once per week	39 (57.4%)	38 (33.0%)	24 (30.8%)	101 (38.7%)

**Fig. 2:**
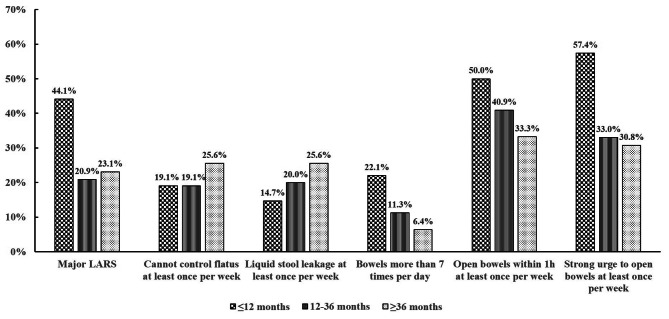
Incidences of 5 score components of LARS in three groups of different time to assessment. The incidences of cases who could not control flatus or had liquid stool leakage did not decrease significantly with time passage postoperatively. In contrast, in accord with decreasing incidence of major LARS, incidences of cases with bowels more than 7 times per day, open bowels within 1 h and strong urge to open bowels at least once per week decreased significantly in the group of 12–36 months when compared to those within 12 months

As shown in Fig. 2, the incidences of cases who could not control flatus or had liquid stool leakage did not decrease significantly with time passage postoperatively. In contrast, in accord with decreasing incidence of major LARS, incidences of cases with bowels more than 7 times per day, open bowels within 1 h and strong urge to open bowels at least once per week decreased significantly in the group of 12–36 months when compared to those within 12 months.

### Analysis of covariates and major LARS

As shown in Table 3, variables of potential risk factors for major LARS were analyzed for further multivariable analysis. Among them, sex (*P* = 0.783), BMI (*P* = 0.318), N-stage (*P* = 0.650), hyperlipemia (*P* = 0.458) and neoadjuvant therapy (*P* = 0.990) were all dropped from the model selection process. The remaining variables with *P* value less than 0.25 were retained for the multivariable analysis.

**Table 3:** Analysis of potential risk factors for major LARS

Variable	LARS		*P* value
	No (n = 149)	Major (n = 62)	
Sex			0.783
Male	98	42	
Female	51	20	
Age (years)	60.4 ± 10.6	63.5 ± 10.2	0.053
<60	69	23	0.219
≥60	80	39	
BMI (kg/m^2^)	22.47 ± 2.71	22.92 ± 3.37	0.318
T-stage			0.041
T_1 − 2_	47	11	
T_3 − 4_	102	51	
N-stage			0.650
N_0_	101	44	
N_1 − 2_	48	18	
Tumor height	8.59 ± 3.74	7.56 ± 3.11	0.058
≤5 cm	39	19	0.155
5-10 cm	76	36	
≥10 cm	34	7	
Hyperlipemia			0.458
Yes	10	6	
No	139	139	
Diabetes			0.161
Yes	14	10	
No	135	52	
Protective stoma			0.012
Yes	23	19	
No	126	43	
Neoadjuvant therapy			0.990
Yes	53	22	
No	96	40	

**Fig. 3:**
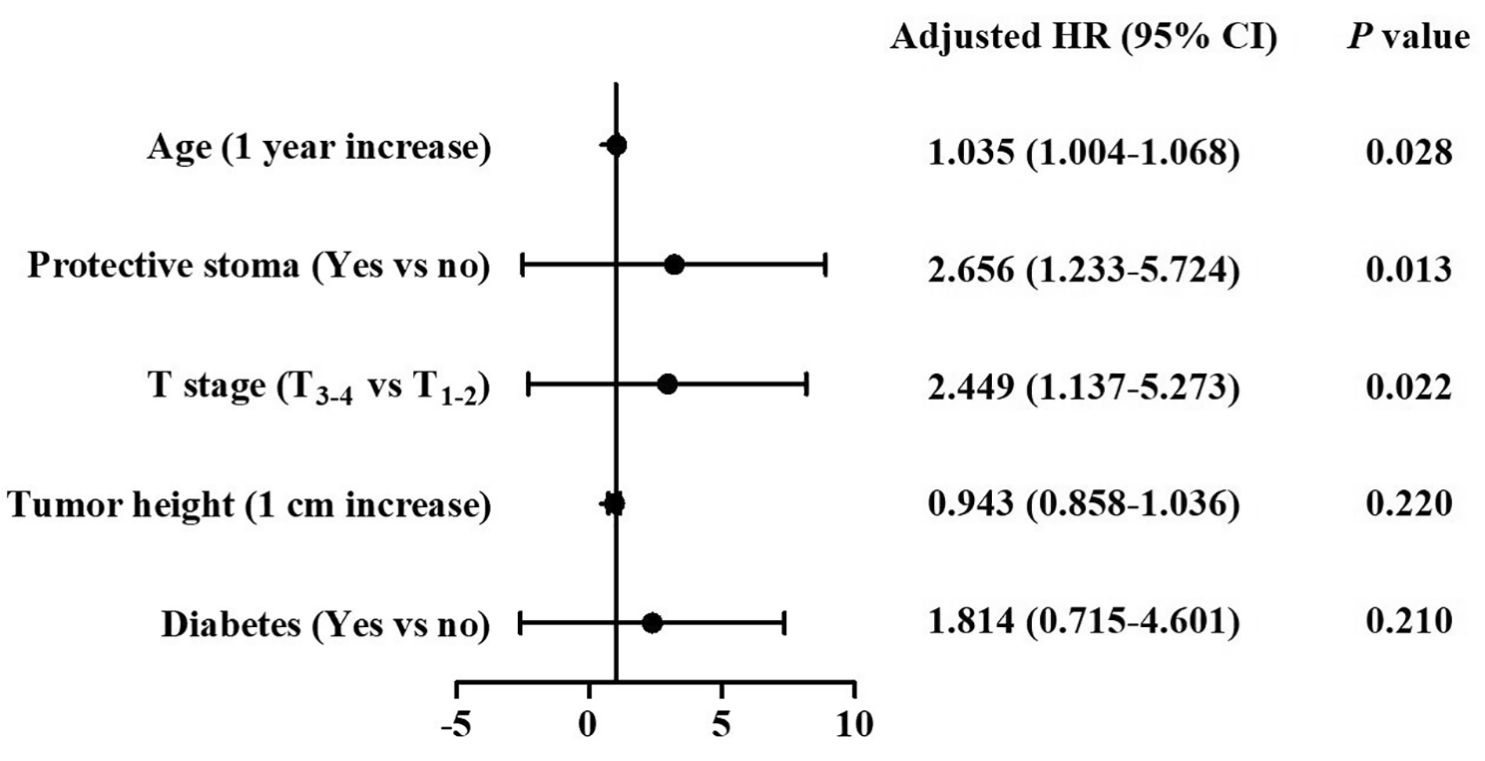
The multivariable analysis of risk factors for major LARS. Age, protective stoma, T-stage, tumor height and diabetes were included in the multivariable model. Age (1 year increase) was associated with a 1.035 (95% CI: 1.004–1.068) increase in the odds of major LARS. Protective stoma and advanced T-stage (T_3 − 4_) had an increase in the odds of major LARS at 2.656 (95% CI: 1.233–5.724) and 2.449 (95% CI: 1.137–5.273) compared to no protective stoma and local T-stage (T_1 − 2_), respectively. Tumor height from the anal verge (1 cm decrease) and comorbid diabetes had an increase in the odds of major LARS, while the differences were not significant

As shown in Fig. 3, age, protective stoma, T-stage, tumor height and diabetes were included in the multivariable model. The result revealed that 1 year increase in age was associated with a 1.035 (95% CI: 1.004–1.068) increase in the odds of major LARS. Protective stoma and advanced T-stage (T_3 − 4_) had an increase in the odds of major LARS at 2.656 (95% CI: 1.233–5.724) and 2.449 (95% CI: 1.137–5.273) compared to no protective stoma and local T-stage (T_1 − 2_), respectively. Although tumor height from the anal verge (1 cm decrease) and comorbid diabetes had an increase in the odds of major LARS, the differences were not statistically significant.

### Results of satisfaction survey

As shown in Table 4, the satisfaction survey contained 6 questions. Incidence of subjective feeling of defecation disorder was consistent with severity of LARS. Totally 87.3% patients with defecation disorder complained their symptoms to the doctors and 84.5% doctors provided treatments for them. Only 36.8% patients with defecation disorder thought they benefited from these treatments.

**Table 4:** Contents and results of satisfaction survey

Contents	Results					
Q1: Do you have trouble in defecation?	Yes		No		Unclear	
No LARS (n = 138)	10 (7.2%)		123 (89.1%)		5 (3.6%)	
Minor LARS (n = 51)	36 (70.6%)		13 (25.5%)		2 (3.9%)	
Major LARS (n = 72)	72 (100%)		0		0	
If yes for Q1, answer Q2 & Q3 (n = 118)						
Q2: How serious are you affected by defecation disorder?	Strong 81 (68.6%)		Moderate 17 (14.4%)		Minor 20 (16.9%)	
Q3: Have you complained to your doctor about your symptoms?	Yes 103 (87.3%)		No 9 (7.6%)		Unclear 6 (5.1%)	
If yes for Q3, answer Q4 (n = 103)						
Q4: Have your doctors provided treatments for your symptoms?	Yes 87 (84.5%)		No 8 (7.8%)		Unclear 8 (7.8%)	
If yes for Q4, answer Q5& Q6 (n = 87)						
Q5: What kinds of treatments do your doctors provided for you? *	PFR n = 15	SNS n = 2	Drugs n = 11	TCM n = 21	Mixed n = 33	Others n = 5
Q6: Do you think your doctors’ treatments work for you?	Yes 32 (36.8%)		No 35 (40.2%)		Unclear 20 (23.0%)	

*PFR: Pelvic floor rehabilitation; SNS: Sacral nerve stimulation; Drugs: Serotonin (5-HT3) receptor antagonists; TCM: Traditional Chinese medicine; Mix: More than one treatment; Others: Others or unclear

## Discussion

LARS is a common manifestation that largely impacts QoL of patients undergoing LAR. However, the incidence of LARS variates in different studies [5, 9, 10]. In this study, we included patients undergoing laparoscopic LAR. The incidence of LARS and major LARS was 47.1% and 27.6% respectively, both lower than those of previous studies [12]. We considered it might be related to the following reasons. First, cases of postoperative anastomotic leakage and neoadjuvant radiotherapy were not included in this study, and previous studies have revealed that these factors significantly increase incidence of LARS [10, 13]. Second, all cases undergoing laparoscopic LAR in this study were performed by a same experienced surgical team. This surgical team are especially good at pelvic autonomic nerve preservation and have proposed a new surgical approach for total mesorectal excision (TME). This innovative TME surgical approach (iTME) has been proved a better effect on preservation of pelvic autonomic nerve (PAN) and postoperative urogenital function [14, 15]. In theory, better preservation of PAN may present less internal anal sphincter dysfunction and decreasing of anal canal sensation, which have been proved to be key pathophysiology of LARS [16–18]. However, there is still lack of direct and solid evidence that iTME has a protective effect on internal anal sphincter function and anal canal sensation. Thus, further study is in urgent need to investigate the effect of iTME on prevention of LARS.

In this study, most doctors provided treatments for patients with defecation disorder. However, previous study reported that although LARS had a large impact on QoL of patients undergoing LAR, it seemed that rectal cancer specialists did not pay enough attention to it and even did not have a thorough understanding of which bowel dysfunction symptoms truly mattered to patients, nor how these symptoms affected QoL [19]. One important reason may be that it is widely considered that the incidence and severity of LARS could be ameliorated with time postoperatively [20]. In this study, the incidences of LARS and major LARS within 12 months postoperatively were both higher than those within 12–36 months, which was consistent with previous study that symptoms of LARS could be relieved between 12 and 24 months postoperatively [20]. However, similar with previous study [10], this study also revealed that the incidence of LARS and major LARS did not decrease after 36 months postoperatively. This result suggests that rectal cancer specialists should pay more attention to prevention of LARS for patients undergoing LAR.

The LARS questionnaire is composed of 5 items, which indicates five components of bowel dysfunction: incontinence to flatus, incontinence to stool, frequency, stool clustering and urgency. The satisfaction survey in this study revealed that the incidence of subjective feeling of defecation disorder was consistent with severity of LARS, suggesting that LARS questionnaire was objective to evaluate the defecation status of patients. A previous systematic review revealed the variety of instruments used to calculate postoperative bowel dysfunction and highlighted the variable symptoms of LARS, and demonstrated that the most common symptoms were incontinence to stool (97.0%), frequency (71.8%) and incontinence to flatus (67.5%) [21]. However, our study revealed that the most common symptoms were stool clustering (41.0%) and urgency (38.7%). A recent study demonstrated a similar result with our study [10]. This inconsistent result indicates that LARS is much more complicated than we expected. Some studies stated that major LARS was not restricted to patients undergoing LAR, while it could also occur in the general population, where the prevalence is up to 18.8% in 50–79 years old women [22]. Thus, the LARS score may be misinterpreted without determining a baseline [23]. Nevertheless, the LARS score is still the most objective and widely applied instruments for calculating bowel function after LAR. In this study, unlike the symptoms of stool clustering and urgency, the symptoms of incontinence to stool and incontinence to flatus did not ameliorate with time postoperatively, suggesting that we should focus more on treatments for these symptoms and thus better reduce severity of LARS.

Although there are kinds of treatments for LARS, the therapeutic effect is not satisfying. In this study, only 36.8% of patients with defecation disorder considered they benefited from the doctors’ treatments. Thus, figuring out the risk factors for LARS is extremely important for early intervention and thus reduce incidence and severity of LARS. In 2018, Battersby et al. [24] demonstrated some key predictive factors for LARS, containing age (at surgery); tumour height, total versus partial mesorectal excision, stoma and preoperative radiotherapy. This team thus developed a nomogram and online tool called Pre-Operative LARS score (POLARS) to predict bowel dysfunction severity prior to anterior resection. In this study, we also proved age and protective stoma as key risk factors for major LARS. It is reasonable that aging can lead to higher incidence of postoperative internal anal sphincter dysfunction and decreasing in anal canal sensation, thus cause major LARS. For protective stoma, a recent meta-analysis study also revealed that protective ileostomy was associated with higher risk of major LARS [25]. A Previous study has demonstrated that ileostomy reversal within 6 months was protective against major LARS, while reversal after 1 year was associated with increased risk of major LARS [26]. In our center, we routinely performed ileostomy reversal within 3 months after initial surgery. In addition, pelvic floor muscle training (PFMT) was routinely performed for patients with protective ileostomy before ileostomy reversal in our center. As one of the standard techniques for the treatment of fecal incontinence, PFMT reduces leakage by improving the structural support, timing, and strength of automatic contractions, thus may help reduce incidence and severity of LARS when assessment was performed after ileostomy reversal [27]. However, the incidence of major LARS was still higher in cases of protective stoma, suggesting that more interventions should be performed for these patients before ileostomy reversal. Pathological advanced T-staging also presented as a risk factor for major LARS in this study. Compared with T_1 − 2_ patients, cases of advanced T-staging may require an extended excision and thus more impair internal anal sphincter and pelvic structure, which leads to occurrence of LARS.

There were some contradictory results compared with previous studies. In this study, although decrease of tumor height and comorbid diabetes revealed an increase in the odds of major LARS, the difference was not statistically significant. Previous study revealed that patients with diabetes were 3.7 times more likely to experience major LARS than those without diabetes [23]. We considered that severity of diabetes may decide the risk of major LARS. Patients with diabetic peripheral neuropathy (DPN) may be more likely to be associated with major LARS, however, there were only two cases of DPN in this study. The relationship of severity of diabetes with major LARS should be further investigated. For tumor height, it is reasonable that resection of low rectal cancer leads to a worse postoperative bowel function, due to greater reduction of rectal reservoir capacity and rectal ampulla dysfunction [28]. In our center, transanal total mesorectal excision (TaTME) is in prior for low rectal cancer with difficulty in performing traditional LAR. TaTME has been reported to be associated with more severe bowel dysfunction than traditional approaches to rectal cancer [29]. In this study, cases of TaTME were excluded and this led to less cases of low rectal cancer included. This may explain why tumor height did not indicate as a risk factor for major LARS significantly in this study.

There are some novel findings in this study. First, we proved that incidence of LARS was highest within 12 months postoperatively, decreased within 12–36 months, but could not be alleviated anymore 36 months later. This result strongly indicated that effective therapy for LARS were necessary, and early interventions may be important to improve the therapeutic effect. Second, we set a questionnaire of satisfaction survey for patients, and revealed that only a few proportions of patients with defection difficulty considered they benefited from the doctors’ treatment, suggesting that the therapeutic effect of LARS was still greatly unsatisfying. A comprehensive therapeutic strategy should be furthered investigated. Third, unlike the symptoms of stool clustering and urgency, the symptoms of incontinence to stool and incontinence to flatus did not ameliorate with time postoperatively in this study, suggesting that we should focus more on treatments for these symptoms and thus better reduce severity of LARS. Last, we proved that protective ileostomy was associated with higher risk of major LARS. Thus, either intervention before ileostomy reversal, like pelvic floor muscle training, or early ileostomy reversal may be useful for reducing incidence of LARS.

## Conclusion

In conclusion, this study reveals that LARS frequently occurred for patients undergoing laparoscopic LAR, while the therapeutic effect is not satisfying. Although partial symptoms of LARS may be alleviated with passage of postoperative time, incidence and severity of LARS could not be reduced anymore after 36 months postoperatively. Thus, rectal cancer specialists should pay more attention to it. Elder, advanced T-stage and patients with protective stoma suffer more frequently from major LARS postoperatively, thus more interventions should be scheduled to decrease incidence and severity of LARS.

## Data Availability

The datasets of the current study are available from the corresponding author upon reasonable request.
